# Stereotactic body radiotherapy for organ-confined prostate cancer

**DOI:** 10.1186/1471-2490-10-1

**Published:** 2010-02-01

**Authors:** Alan J Katz, Michael Santoro, Richard Ashley, Ferdinand Diblasio, Matthew Witten

**Affiliations:** 1 Winthrop University Hospital, 264 Old Country Road, Mineola, NY 11501, USA

## Abstract

**Background:**

Improved understanding of prostate cancer radiobiology combined with advances in delivery of radiation to the moving prostate offer the potential to reduce treatment-related morbidity and maintain quality of life (QOL) following prostate cancer treatment. We present preliminary results following stereotactic body radiotherapy (SBRT) treatment for organ-confined prostate cancer.

**Methods:**

SBRT was performed on 304 patients with clinically localized prostate cancer: 50 received 5 fractions of 7 Gy (total dose 35 Gy) and 254 received 5 fractions of 7.25 Gy (total dose 36.25 Gy). Acute and late toxicity was assessed using the Radiation Therapy Oncology Group scale. The Expanded Prostate Cancer Index Composite questionnaire was used to assess QOL. Prostate-specific antigen response was monitored.

**Results:**

At a median 30-month (26 - 37 month, range) follow-up there were no biochemical failures for the 35-Gy dose level. Acute Grade II urinary and rectal toxicities occurred in 4% of patients with no higher Grade acute toxicities. One Grade II late urinary toxicity occurred with no other Grade II or higher late toxicities. At a median 17-month (8 - 27 month, range) follow-up the 36.25 Gy dose level had 2 low- and 2 high-risk patients fail biochemically (biopsy showed 2 low- and 1 high-risk patients were disease-free in the gland). Acute Grade II urinary and rectal toxicities occurred in 4.7% (12/253) and 3.6% (9/253) of patients, respectively. For those patients with a minimum of 12 months follow-up, 5.8% (12/206) had late Grade II urinary toxicity and 2.9% (6/206) had late Grade II rectal toxicities. One late Grade III urinary toxicity occurred; no Grade IV toxicities occurred. For both dose levels at 17 months, bowel and urinary QOL returned to baseline values; sexual QOL decreased by 10%.

**Conclusions:**

The low toxicity and maintained QOL are highly encouraging. Additional follow-up is needed to determine long-term biochemical control and maintenance of low toxicity and QOL.

## Background

Conventional treatments for localized prostate cancer target local control at the potential expense of morbidity and decreased quality of life. Urinary function impairment has been reported to occur in 5-28% of patients at 2 years after radical prostatectomy (RP) and in 2-14% of patients at 2 years after external beam radiation therapy (EBRT) [1,2]. Bowel distress is reported in 3 - 21% of RP and 8 - 37% of EBRT patients 2 years after treatment [2]. Erectile dysfunction has been reported at rates of 51-82% and 30-51% two-years following RP and EBRT, respectively [2-4]. Sexual quality of life estimates show similar results for these treatments [1]. Indeed, the rate of such complications, and the extent to which they reduce the quality of life of prostate cancer patients, contributed to a recent recommendation from the United States Preventive Services Task Force (USPTF) against routine prostate-specific antigen (PSA) screening for prostate cancer in men aged 75 or older [5].

Advances in targeted radiation delivery and a modern understanding of the radiobiology of prostate cancer suggest approaches to controlling prostate cancer while decreasing treatment-related toxicity. Radiobiologically, slowly proliferating prostate cancer cells are thought to have a low α/β ratio; a recent review of 17 studies estimated an average α/β ratio of 1.85 Gy [6]. This low α/β ratio suggests that prostate cancer has high sensitivity to dose per fraction, which suggests that a hypofractionated radiation delivery regimen, with a large radiation dose delivered in a smaller number of fractions, may be advantageous.

The first reported hypofractionated radiation therapy treatments for prostate cancer occurred in the early 1960's [7]. These treatments, delivering 6 fractions of 6 Gy to a total dose of 36 Gy, were motivated by resource limitations rather than radiobiology. Nevertheless, two decades of follow-up has confirmed that this regimen led to favorable local response, survival, and safety over the long term. Subsequently, hypofractionated prostate cancer treatment has been performed with EBRT in per-fraction doses ranging from 2.5 - 3.1 Gy [8-11], with brachytherapy (BT) in per-fraction doses of 5.5 - 11.5 Gy[12,13], and with linac-based stereotactic body radiotherapy (SBRT) using 5 fractions of 6.7 Gy [14]. In a recent paper King et al. reported a median 33-month follow-up for patients that received 5 fractions of 7.25 Gy (total dose 36.25 Gy). They reported no biochemical failure with early and late toxicity profiles no worse than conventional EBRT [15]. Thus, in relatively short-term follow-up, hypofractionated treatment of prostate cancer can result in effective biochemical control while maintaining low rectal and bladder toxicities.

Technological advances have allowed precise targeting and delivery of radiation to the moving prostate while sparing normal tissues. This suggests the potential to reduce treatment-related morbidity and maintain quality of life following prostate cancer treatment. In this report, we present preliminary biochemical control results on the treatment of 304 low-, intermediate-, and high-risk prostate cancer patients using SBRT, with particular attention to urinary, rectal and sexual toxicities and their corresponding impact on patient quality of life.

## Methods

### Patient population

Data were prospectively collected for all clinically localized prostate cancer patients that were treated with SBRT at Winthrop University Hospital in Mineola, NY between April 2006 and July 2008. The 304 patients all had adenocarcinoma of the prostate; 280 (92.2%) patients presented with clinical stage T1cN0 M0 and 24 (7.8%) patients presented with clinical stage T2aN0 M0 as determined by physical exam, bone scan and CT scans. The median PSA at presentation was 5.8 ng/ml (range 0.7 - 27.3 ng/ml). Table 1 details the patient characteristics. All patients signed consent statements and were informed of the potential risks involved with this treatment. Institutional IRB-approval was obtained on the treatment protocol.

**Table 1:** Patient characteristics at diagnosis.

Age at diagnosis	Years	
Mean (range)	69.2 (45 - 88)	

**Age at diagnosis**	**Number of Patients**	**Percent of Patients**

45-49	1	0.3

50-54	7	2.3

55-59	23	7.6

60-64	35	11.5

65-70	54	17.8

70-74	80	26.3

75-79	54	17.8

80-84	36	11.8

85-88	14	4.6

**PSA level at diagnosis**	**ng/mL**	

Mean (range)	6.08 (0.7 to 27.7)	

Median	5.8	

**PSA level at diagnosis**	**Number of Patients**	**Percent of Patients**

<4 ng/mL	59	19.4

4-10 ng/mL	203	66.8

>10-20 ng/mL	40	13.2

>20 ng/mL	2	0.7

**Clinical Stage**	**Number of Patients**	**Percent of Patients**

T1cN0 M0	280	92.1

T2aN0 M0	24	7.9

**Gleason Score**	**Number of Patients**	**Percent of Patients**

= 6	222	73

= 7	70	23

> 8	12	4

**Hormone Treatment**	**Number of Patients**	**Percent of Patients**

No	247	81.3

Yes	57	18.8

**Risk Assessment: Criteria**	**Number of Patients**	**Percent of Patients**

Low Risk: Gleason Score ≤ 6 *and *PSA ≤ 10 ng/ml.	211	69.4

Intermediate Risk: Gleason = 7 *or *PSA>10 *and *PSA < 20	81	26.6

High Risk: Gleason ≥ 8 *or *PSA > 20	12	3.9

### Hormone therapy

Fifty-seven patients received neoadjuvant hormonal therapy. Of those patients, 29(51%) received hormone therapy for three months or less as it was generally stopped at the time of consultation. The remaining 28 patients (49%) received hormone therapy for up to one year at the discretion of the patient's urologist.

### Treatment planning and delivery

Image-guided SBRT was delivered to all patients using the CyberKnife (Accuray Inc., Sunnyvale, CA) with Multiplan inverse treatment planning and motion tracking of internal fiducial seeds. A detailed description of the CyberKnife system can be found elsewhere [16].

Approximately two weeks before treatment planning, four fiducial seeds were placed transperineally in each patient to allow for motion tracking during treatment. The implanted seeds were positioned with two at the prostate apex and two at the base. After allowing time for possible seed migration, treatment planning was performed prior to the treatment day using a CT scan (1.5-mm cuts), with MRI fusion where feasible. All pretreatment imaging was performed with the patient in the same position used for treatment delivery. For low-risk patients, the prostate alone was the gross target volume (GTV). For intermediate- to high-risk patients, the proximal half of the seminal vesicles was added to the GTV if the Gleason Score > 6 and the PSA > 15 ng/ml. Following delineation of the GTV, a margin was added to create the planning target volume (PTV). For low- and intermediate-risk patients, the margin was 5 mm throughout except posteriorly by the rectum where a 3-mm margin was used. For high-risk patients, an 8-mm margin was added on the involved side. All patients had the bladder, prostate, rectum, seminal vesicles and penile bulb contoured, but the urethra was not identified.

**Figure 1:**
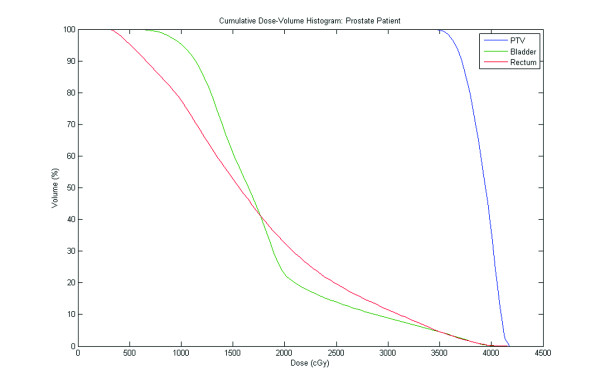
**Dose volume histogram for PTV, bladder and rectum**.

SBRT was delivered at two dose levels. The first 50 treated patients (16%) received a total dose of 35 Gy in 5 fractions of 7 Gy to cover at least 96% of the PTV. The subsequent 254 patients (84%) received a total dose of 36.25 Gy in 5 fractions of 7.25 Gy to cover at least 96% of the PTV. The dose was increased when researchers reported [17] using a higher dose with acceptable toxicity. The mean number of beams was 152 (range 140 - 170). The mean D50 to the bladder and rectum was 43% and 41% of the prescribed dose, respectively. Figure 1 presents a representative dose volume histogram.

Treatments were performed on five consecutive days. The morning of each treatment patients had a bowel prep including Dulcolax^® ^(Boehringer Ingelheim, Germany) and a Fleet Enema. In addition, at least 15 - 20 minutes before treatment all patients received 1500 mg of amifostine (MedImmune, LLC Gaithersburg, MD) mixed in saline instilled into the rectum.

### Follow-up schedule and toxicity assessment

All patients were seen for follow-up three weeks after final treatment, again four months later, and every six months thereafter. Toxicity was assessed at every follow-up visit using the Expanded Prostate Cancer Index Composite (EPIC) questionnaire [18] and the Radiation Therapy Oncology Group (RTOG) urinary and rectal toxicity scale [19]. Acute toxicity was defined as those events that presented and resolved within the first 5 months following treatment. PSA was assessed by the referring urologist 6 months after treatment and every 6 months thereafter. Biochemical failure was the end point of the study, using the Phoenix (nadir + 2) biochemical failure definition [20].

## Results

### Follow-up

The median follow-up for patients receiving the lower dose (35 Gy) was 30 months (range 26 - 37 months). The median follow-up for patients receiving the higher dose (36.25 Gy) was 17 months (range 8 - 27 months). Three patients in the 35-Gy dose level died and three in the 36.25-Gy dose level died, none of prostate cancer.

### Toxicity

The 5-month toxicity follow-up has occurred for all patients except one who died from causes other than prostate cancer at 4 months. Thus, we have acute toxicity profiles for 303 patients. Table 2 presents these acute urinary and rectal toxicities on the RTOG scale broken down by treatment dose. No patients experienced any Grade III or IV acute complications. Less than 5% of patients (14/303) experienced any acute Grade II urinary or rectal toxicity.

**Table 2:** Acute bladder/rectal toxicity using RTOG scoring after prostate treatment using the 35 and 36.25 Gy doses.

		RTOG grade % (number) of patients			
	**Total Dose**	**0**	**I**	**II**	**III & IV**

**Acute Urinary**	35 Gy	24% (12)	72% (36)	4% (2)	-
	
	36.25 Gy	20.2% (51)	75.1% (190)	4.7% (12)	-

**Acute Rectal**	35.00 Gy	20% (10)	76% (38)	4% (2)	-
	
	36.25 Gy	21.7% (55)	74.7% (189)	3.6% (9)	-

Table 3 presents late urinary and rectal toxicities broken down by dose for those patients with a minimum follow-up of 12 months. For the 35-Gy dose level, 2 patients died before their 12-month follow-up. Thus, while follow-up for all other patients extends to 26 months the late toxicity results only include 48 patients. For the 36.25-Gy dose level, 2 patients died before their 12-month follow-up and 46 patients have not yet reached their 12-month follow-up. Late urinary Grade II complications were observed in 5.1% of patients (13/256) and late rectal Grade II complications were observed in 2.3% of patients (6/256). One late Grade III urinary toxicity occurred in the 36.25 Gy dose level. No significant differences in complication rates were observed for patients receiving the 35 Gy or 36.25 Gy doses.

**Table 3:** Late bladder/rectal toxicity using RTOG scoring after prostate treatment using the 35 and 36.25 Gy doses for those patients with a minimum 12 month follow-up

		RTOG grade % (number) of patients				
	**Total Dose**	**0**	**I**	**II**	**III**	**IV**

**Late Urinary**	35 Gy	94% (45)	4% (2)	2% (1)	-	-
	
	36.25 Gy	88.9% (183)	4.8% (10)	5.8% (12)	0.5% (1)	-

**Late Rectal**	35 Gy	95.8% (46)	4.2% (2)	-	-	-
	
	36.25 Gy	91.8% (189)	5.3% (11)	2.9% (6)	-	-

### Quality of life

**Figure 2:**
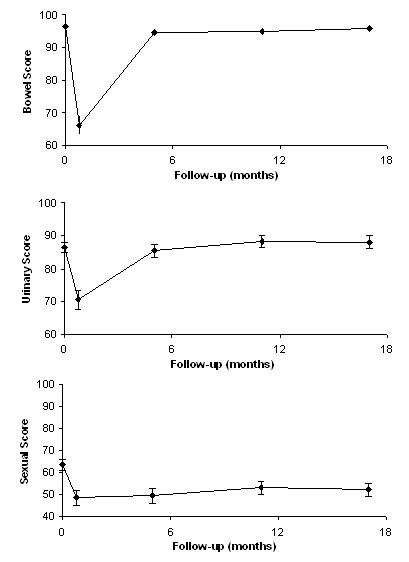
**EPIC Quality of Life scores over time for bowel, urinary and sexual function**. All patients initially completed the EPIC questionnaire, at 3 weeks 264 of 304 (86.84%) patients responded, at 5 months 203 of 304 (66.78%) responded, at 11 months 175 of 272 (64.34%) responded, and at 17 months 145 of 192 (75.53%) responded. Error bars represent 95% confidence intervals. Higher scores represent a better quality of life.

All patients completed the initial EPIC questionnaire prior to treatment. For subsequent time points the number of patients varied depending on the number that reached each follow-up time point and the number that completed the questionnaires. Figure 2 shows the EPIC scores for bowel, urinary and sexual quality of life (QOL) along with patient response rates. Bowel and urinary QOL scores decreased initially before returning to baseline values. For sexual QOL, an overall decrease of 10% in the QOL score was observed. To further examine sexual QOL, we verbally screened patients that were potent prior to treatment (n = 228) to determine if they remained potent. At a median 18 months follow-up (range 7-37 months) 87% percent (198/228) stated they maintained potency, either with or without erectile dysfunction medication.

### Biochemical control, PSA response and PSA nadir

**Figure 3:**
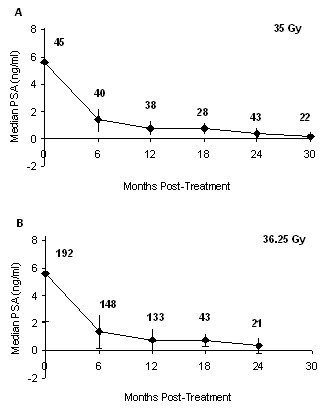
**Median PSA levels (ng/ml +/- 1 standard deviation) for patients who did not receive ADT and who have a minimum follow-up of 12 month in the (A) 35 Gy dose level and (B) 36.25 Gy dose level**.

As with late toxicity, we have limited our PSA analysis to those patients with a minimum 12-month follow-up. In addition, we have excluded the patients who received ADT treatment. The mean PSA decreased after treatment as shown in Figure 3 which presents the PSA response for the two different dose levels. We note that the number of patients for which PSA is available at each time point varied depending on the number that reached each follow-up time point and the number that completed their PSA blood work. Table 4 presents the number of patients achieving the specified PSA nadir thresholds over time.

**Table 4:** Percent of patients who did not receive hormone therapy with at least 12 months follow-up achieving specified PSA nadir threshold over time.

	Percent (Number) of patients achieving specified PSA nadir				
**35 Gy**	**6 months** **(40 patients)**	**12 months** **(38 patients)**	**18 months** **(28 patients)**	**24 months** **(43 patients)**	**30 months** **(32 patients)**

**< 1 ng/mL**	48% (19)	55% (21)	57% (16)	88% (38)	88% (28)

**< .5 ng/mL**	15% (6)	37% (14)	46% (13)	65% (28)	75% (24)

					

**36.25 Gy**	**6 months** **(148 patients)**	**12 months** **(133 patients)**	**18 months** **(43 patients)**	**24 months** **(21 patients)**	

**< 1 ng/mL**	35% (53)	45% (60)	76% (33)	81% (17)	

**< .5 ng/mL**	19% (29)	26% (34)	51% (22)	66% (14)	

A chi-square test of independence was performed between the 35 Gy and 36.25 Gy. For both the < 1 ng/ml (χ2 = 5.794, p = .122) and < .5 ng/ml (χ2 = 2.259, p = .520) cases the chi-square test shows no significant difference is present between 35 Gy and 36.25 Gy.

Two low-risk and two high-risk patients, all treated with the higher dose, failed biochemically. None of the intermediate-risk patients failed biochemically. The two low-risk patients that failed biochemically were both shown free of disease in the gland by 12-core biopsy. One of the two high-risk patients that failed was biopsy proven to be recurrent in the gland with 4 out of the 12 cores showing a Gleason Score of 10. A PSA bounce (defined as an increased PSA > 0.2 ng/mL followed by a decreased PSA to previous value or lower) occurred in 16% (37/237 of the patients at a median of 18 months. The median bounce was 0.35 ng/ml (range 0.2 - 1.08 ng/ml).

## Discussion

### Acute toxicity

A variety of RTOG graded toxicity rates have been reported for both conventionally fractionated EBRT and intensity modulated radiation therapy (IMRT) treatment of prostate cancer. Michalski et al. and Peeters et al. examined delivery of 78 Gy in 2-Gy fractions with reported rates of 36-41% for Grade II and 4-14% for Grade III acute urinary toxicity [21,22]. For acute rectal toxicity, Peeters et al. reported 44% Grade II and 5% Grade III toxicity [22]. Bechendorf et al. applied a dose of 80 Gy in 2-Gy fractions with 31% Grade II and 5% Grade III acute urinary toxicity; acute rectal toxicity rates were 29% for Grade II and 2% for Grade III [23]. Zietman et al. applied a dose of 79.2 Gy using a combination of EBRT and protons with 49% Grade II, 1% Grade III, and 1% Grade IV acute urinary toxicity; acute Grade II rectal toxicity was 57%. In a study of IMRT in which 81-85 Gy were delivered in 1.8-Gy fractions, 28% Grade II and 0.1% Grade III acute urinary toxicity was reported; acute Grade II rectal toxicity was 4.5%. In comparison, assuming an α/β = 1.5 Gy, our dose regimens equal approximately 91 Gy and 96 Gy, respectively, at 1.8 Gy per fraction. Thus, although our therapeutic doses were higher than the above studies our observed rate of acute urinary and rectal toxicity was lower, with less than 5% of patients experiencing any acute Grade II urinary or rectal toxicity and none experiencing any higher grade acute toxicity.

### Late toxicity

Our results to date for hypofractionated SBRT of localized prostate cancer when administered with rectal amifostine also indicate less late toxicity than that previously published for hypofractionated SBRT and conventionally fractionated EBRT. King et al. treated 41 patients with SBRT to a total dose of 36.25 Gy in 5 fractions of 7.25 Gy [15]. At a median 33-month follow-up they reported late Grade II urinary toxicity in 24% of patients whereas we observed 2% Grade II urinary toxicity at 30 months follow-up for the 35 Gy dose level. At 17 months median follow-up for the 36.25 Gy dose level we observed 5.8% Grade II late urinary toxicity and one late Grade III urinary toxicity. As discussed by King et al. these late urinary toxicities may increase over time, therefore, the durability of our observed late urinary toxicity requires additional follow-up.

For late bowel toxicity, King et al. observed 15% Grade II toxicity at a median 33-month follow-up [15]. An EBRT dose-escalation trial [24] in which 78 Gy was delivered in 2-Gy fractions obtained similar late rectal toxicities, with 19% and 7% of patients observing Grade II and Grade III toxicities, respectively, at a median follow-up of 7 years. Similar results have also been reported for EBRT + protons by Zietman et al. [25] with delivery of either 70.2 Gy or 79.2 Gy at a median follow-up of 5.5 years. Our observed bowel toxicity rate at a median 30-month follow-up for the 35 Gy dose level is 4.2% (2/48) for Grade I toxicities with no higher grade toxicities. Rectally administered amifostine prior to EBRT for prostate cancer has resulted in a significant decrease in RTOG Grade II toxicity incidence [26]. Thus, we attribute our low bowel toxicity rate to rectal administration of amifostine prior to delivery of each treatment fraction. Rectal administration of amifostine is easy to perform and has no related toxicity in comparison to intravenous administration [26,27].

The MD Anderson results show that the rate of toxicity for EBRT treatment continued to increase during the first 5 years following treatment before hitting a plateau [24]. However, other studies have shown late rectal toxicity to occur less frequently as time progresses with comparable toxicity-free survival at 3 versus 5 years [25,28]. Thus, while our rectal toxicity rates may increase over time, the low rectal toxicity we have observed to date is highly encouraging.

### Quality of life

In a large study of QOL, Sanda et al. compared QOL in patients treated with RP (n = 603), EBRT (n = 292) and low-dose rate BT (n = 306) using, among other measures, EPIC scores [1]. Comparison of these results at 24 months to the current studies SBRT results at 17 months suggests that our hypofractionated SBRT approach resulted in an overall similar or potentially better QOL to these other treatment modalities. In the case of the bowel QOL, the Sanda et al. study shows an approximate 10% decrease in EPIC QOL for both EBRT and BT. Our results show a return to pre-treatment QOL that matches those observed for RP, suggesting an improved QOL for SBRT over both EBRT and BT for the bowel. In the case of urinary QOL, the Sanda et al. results show a near return to baseline EPIC values for EBRT and BT, but a significant loss of QOL for prostatectomy [1].

Although our baseline urinary score is slightly lower than those in the Sanda et al. study (86.5% versus approximately 93%), we observed a similar overall recovery to baseline values that exceeded those observed for RT and BT. This improvement of urinary QOL is consistent with the SBRT results observed by King et al. [15]. Lastly, for sexual QOL our results parallel those of RT and BT, showing about a 10% decrease in sexual QOL. Prostatectomy, which had a higher initial baseline, shows an overall significantly worse sexual QOL at 2 years.

The current study reports EPIC results at the 17-month follow-up, with 75% (145/192) of patients completing the EPIC questionnaires (i.e., responses at 17 months are available for 48% of all patients). The comparisons to the Sanda et al. study are based on their EPIC scores at 24 months. Examination of the Sanda results show that, in general, EPIC QOL scores plateau or increase only slightly between the 12-month and 2-year follow-up. Our results show no significant difference between the 11- and 17-month EPIC QOL; whether EPIC QOL at the 2-year follow-up will remain stable will require continued follow-up.

### PSA response & biochemical control

**Figure 4:**
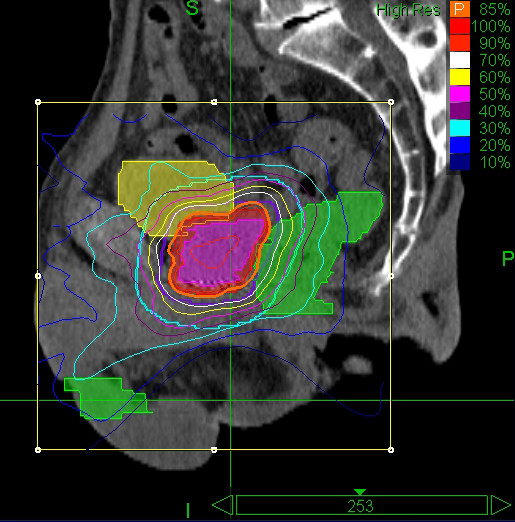
**Representative sagittal view of a treatment plan illustrating dose to testes**.

At a median 30-month follow-up for patients treated with 35 Gy, the reduction in PSA values is very similar to that reported by King et al. [15]. In addition, we observed a similar overall PSA response for patients with a minimum 12-month follow-up independent of overall treatment dose (Figure 3 and Table 4). Although encouraging, it should be noted that a recent commentary by King et al. identified the potential for some beams in CyberKnife prostate treatments to pass through the testicles [29]. We examined 12 patients' treatment plans and found the median D50 testicular dose was 5.28 Gy (range, 3.2 - 11.8 Gy, see Figure 4). King et al. noted that some studies suggest hypogonadism and altered PSA values can occur at doses as low as 2-4 Gy following conformal radiation treatments [30,31]. In contrast, a study of IMRT treatment for prostate cancer found mean doses of 5.4 and 5.1 Gy to the right and left testicles did not significantly alter either testosterone levels or PSA levels [32]. We have not observed any clinical evidence of hypogonadism in any patients. While we acknowledge the testicular dose could alter serum testosterone and PSA values, our results suggest that this did not happen in the current study. Nevertheless, we examine all our treatment plans carefully, and attempt to exclude beams that transit the testicles while maintaining the overall quality of treatment plan. We will also assay testosterone levels as part of our ongoing follow-up.

## Conclusions

Our results show that SBRT of early stage prostate cancer with rectal administration of amifostine can be performed with low acute toxicity. At a median 30-month follow-up for the 35 Gy dose level, the long-term urinary and rectal toxicity are both low. EPIC QOL questionnaires also suggest that urinary, rectal and sexual QOL following SBRT may be comparable, if not better than that for EBRT, BT and RP. Furthermore, at our facility SBRT is less costly (by roughly $15,000US) than IMRT while being much less inconvenient for the patient than a 45-day course of IMRT. Longer term follow-up and additional studies are needed to confirm the durability of biochemical control, toxicity and QOL with SBRT of prostate cancer.

## Competing interests

Dr. Katz has received speaker's honoraria from Accuray, Inc., Sunnyvale CA. The remaining authors declare that they have no competing interests.

## Authors' contributions

AK was responsible for the treatment of the patients, collection of data, interpretation of data and manuscript preparation. MS, RA, FD, and MW were responsible for gathering and interpreting data, manuscript revision and final manuscript approval. All authors read and approved the final manuscript.

## Pre-publication history

The pre-publication history for this paper can be accessed here:

http://www.biomedcentral.com/1471-2490/10/1/prepub

## References

[B1] Sanda MG Dunn RL Michalski J Sandler HM Northouse L Hembroff L Lin X Greenfield TK Litwin MS Saigal CS Mahadevan A Klein E Kibel A Pisters LL Kuban D Kaplan I Wood D Ciezki J Shah N Wei JT Quality of life and satisfaction with outcome among prostate-cancer survivors N Engl J Med 2008 358 12 1250 1261 10.1056/NEJMoa074311 18354103

[B2] Potosky AL Legler J Albertsen PC Stanford JL Gilliland FD Hamilton AS Eley JW Stephenson RA Harlan LC Health outcomes after prostatectomy or radiotherapy for prostate cancer: results from the Prostate Cancer Outcomes Study J Natl Cancer Inst 2000 92 19 1582 1592 10.1093/jnci/92.19.1582 11018094

[B3] Robinson JW Moritz S Fung T Meta-analysis of rates of erectile function after treatment of localized prostate carcinoma Int J Radiat Oncol Biol Phys 2002 54 4 1063 1068 10.1016/S0360-3016(02)03030-4 12419432

[B4] Siglin J Kubicek GJ Leiby B Valicenti RK Time of Decline in Sexual Function After External Beam Radiotherapy for Prostate Cancer Int J Radiat Oncol Biol Phys 2010 76 1 31 35 19395191 10.1016/j.ijrobp.2009.01.070

[B5] Screening for prostate cancer U.S. Preventive Services Task Force recommendation statement Ann Intern Med 2008 149 3 185 191 18678845 10.7326/0003-4819-149-3-200808050-00008

[B6] Dasu A Is the alpha/beta value for prostate tumours low enough to be safely used in clinical trials? Clin Oncol (R Coll Radiol) 2007 19 5 289 301 17517328 10.1016/j.clon.2007.02.007

[B7] Collins CD Lloyd-Davies RW Swan AV Radical external beam radiotherapy for localised carcinoma of the prostate using a hypofractionation technique Clin Oncol (R Coll Radiol) 1991 3 3 127 132 2069876 10.1016/s0936-6555(05)80831-3

[B8] Yeoh EE Holloway RH Fraser RJ Botten RJ Di Matteo AC Butters J Weerasinghe S Abeysinghe P Hypofractionated versus conventionally fractionated radiation therapy for prostate carcinoma: updated results of a phase III randomized trial Int J Radiat Oncol Biol Phys 2006 66 4 1072 1083 16965866 10.1016/j.ijrobp.2006.06.005

[B9] Lukka H Hayter C Julian JA Warde P Morris WJ Gospodarowicz M Levine M Sathya J Choo R Prichard H Brundage M Kwan W Randomized trial comparing two fractionation schedules for patients with localized prostate cancer J Clin Oncol 2005 23 25 6132 6138 10.1200/JCO.2005.06.153 16135479

[B10] Kupelian PA Willoughby TR Reddy CA Klein EA Mahadevan A Hypofractionated intensity-modulated radiotherapy (70 Gy at 2.5 Gy per fraction) for localized prostate cancer: Cleveland Clinic experience Int J Radiat Oncol Biol Phys 2007 68 5 1424 1430 17544601 10.1016/j.ijrobp.2007.01.067

[B11] Livsey JE Cowan RA Wylie JP Swindell R Read G Khoo VS Logue JP Hypofractionated conformal radiotherapy in carcinoma of the prostate: five-year outcome analysis Int J Radiat Oncol Biol Phys 2003 57 5 1254 1259 14630259 10.1016/s0360-3016(03)00752-1

[B12] Demanes DJ Rodriguez RR Schour L Brandt D Altieri G High-dose-rate intensity-modulated brachytherapy with external beam radiotherapy for prostate cancer: California endocurietherapy's 10-year results Int J Radiat Oncol Biol Phys 2005 61 5 1306 1316 15817332 10.1016/j.ijrobp.2004.08.014

[B13] Martinez A Gonzalez J Spencer W Gustafson G Kestin L Kearney D Vicini FA Conformal high dose rate brachytherapy improves biochemical control and cause specific survival in patients with prostate cancer and poor prognostic factors J Urol 2003 169 3 974 979 discussion 979-980 10.1097/01.ju.0000052720.62999.a9 12576825

[B14] Madsen BL Hsi RA Pham HT Fowler JF Esagui L Corman J Stereotactic hypofractionated accurate radiotherapy of the prostate (SHARP), 33.5 Gy in five fractions for localized disease: first clinical trial results Int J Radiat Oncol Biol Phys 2007 67 4 1099 1105 17336216 10.1016/j.ijrobp.2006.10.050

[B15] King CR Brooks JD Gill H Pawlicki T Cotrutz C Presti JC Jr Stereotactic Body Radiotherapy for Localized Prostate Cancer: Interim Results of a Prospective Phase II Clinical Trial Int J Radiat Oncol Biol Phys 2009 73 4 1043 1048 18755555 10.1016/j.ijrobp.2008.05.059

[B16] Romanelli P Schaal DW Adler JR Image-guided radiosurgical ablation of intra- and extra-cranial lesions Technol Cancer Res Treat 2006 5 4 421 428 16866572 10.1177/153303460600500410

[B17] Hara W Patel D Pawlicki T Cotrutz C Presti J King C Hypofractionated stereotactic radiotherapy for prostate cancer: early results Int J Radiat Oncol Biol Phys 2006 66 S324 325

[B18] Wei JT Dunn RL Litwin MS Sandler HM Sanda MG Development and validation of the expanded prostate cancer index composite (EPIC) for comprehensive assessment of health-related quality of life in men with prostate cancer Urology 2000 56 6 899 905 10.1016/S0090-4295(00)00858-X 11113727

[B19] National Institutes of Health Common toxicity criteriaversion 2.0 edn 1998 National Institutes of Health

[B20] Roach M Hanks G Thames H Jr Schellhammer P Shipley WU Sokol GH Sandler H Defining biochemical failure following radiotherapy with or without hormonal therapy in men with clinically localized prostate cancer: recommendations of the RTOG-ASTRO Phoenix Consensus Conference Int J Radiat Oncol Biol Phys 2006 65 4 965 974 16798415 10.1016/j.ijrobp.2006.04.029

[B21] Michalski JM Winter K Purdy JA Parliament M Wong H Perez CA Roach M Bosch W Cox JD Toxicity after three-dimensional radiotherapy for prostate cancer on RTOG 9406 dose Level V Int J Radiat Oncol Biol Phys 2005 62 3 706 713 15936549 10.1016/j.ijrobp.2004.11.028

[B22] Peeters ST Heemsbergen WD van Putten WL Slot A Tabak H Mens JW Lebesque JV Koper PC Acute and late complications after radiotherapy for prostate cancer: results of a multicenter randomized trial comparing 68 Gy to 78 Gy Int J Radiat Oncol Biol Phys 2005 61 4 1019 1034 15752881 10.1016/j.ijrobp.2004.07.715

[B23] Beckendorf V Guerif S Le Prise E Cosset JM Lefloch O Chauvet B Salem N Chapet O Bourdin S Bachaud JM Maingon P Lagrange JL Malissard L Simon JM Pommier P Hay MH Dubray B Luporsi E Bey P The GETUG 70 Gy vs. 80 Gy randomized trial for localized prostate cancer: feasibility and acute toxicity Int J Radiat Oncol Biol Phys 2004 60 4 1056 1065 15519775 10.1016/j.ijrobp.2004.05.033

[B24] Kuban DA Tucker SL Dong L Starkschall G Huang EH Cheung MR Lee AK Pollack A Long-term results of the M. D. Anderson randomized dose-escalation trial for prostate cancer Int J Radiat Oncol Biol Phys 2008 70 1 67 74 17765406 10.1016/j.ijrobp.2007.06.054

[B25] Zietman AL DeSilvio ML Slater JD Rossi CJ Jr Miller DW Adams JA Shipley WU Comparison of conventional-dose vs high-dose conformal radiation therapy in clinically localized adenocarcinoma of the prostate: a randomized controlled trial Jama 2005 294 10 1233 1239 10.1001/jama.294.10.1233 16160131

[B26] Simone NL Menard C Soule BP Albert PS Guion P Smith S Godette D Crouse NS Sciuto LC Cooley-Zgela T Camphausen K Coleman CN Singh AK Intrarectal amifostine during external beam radiation therapy for prostate cancer produces significant improvements in Quality of Life measured by EPIC score Int J Radiat Oncol Biol Phys 2008 70 1 90 95 17855015 10.1016/j.ijrobp.2007.05.057 PMC2267374

[B27] Athanassiou H Antonadou D Coliarakis N Kouveli A Synodinou M Paraskevaidis M Sarris G Georgakopoulos GR Panousaki K Karageorgis P Throuvalas N Protective effect of amifostine during fractionated radiotherapy in patients with pelvic carcinomas: results of a randomized trial Int J Radiat Oncol Biol Phys 2003 56 4 1154 1160 10.1016/S0360-3016(03)00187-1 12829154

[B28] Duchesne GM Williams SG Das R Tai KH Patterns of toxicity following high-dose-rate brachytherapy boost for prostate cancer: mature prospective phase I/II study results Radiother Oncol 2007 84 2 128 134 10.1016/j.radonc.2007.05.019 17561293

[B29] King CR Lo A Kapp DS Testicular dose from prostate cyberknife: a cautionary note Int J Radiat Oncol Biol Phys 2009 73 2 636 637 author reply 637 19147028 10.1016/j.ijrobp.2008.09.004

[B30] Daniell HW Clark JC Pereira SE Niazi ZA Ferguson DW Dunn SR Figueroa ML Stratte PT Hypogonadism following prostate-bed radiation therapy for prostate carcinoma Cancer 2001 91 10 1889 1895 10.1002/1097-0142(20010515)91:10<1889::AID-CNCR1211>3.0.CO;2-U 11346871

[B31] Bruheim K Svartberg J Carlsen E Dueland S Haug E Skovlund E Tveit KM Guren MG Radiotherapy for rectal cancer is associated with reduced serum testosterone and increased FSH and LH Int J Radiat Oncol Biol Phys 2008 70 3 722 727 18262088 10.1016/j.ijrobp.2007.10.043

[B32] Yogeswaren ST Teh BS Mai W Childress C McGary JE Grant WH Butler E Radiation dose to testicles and serum testosterone levels in low risk prostate cancer patients undergoing intensity-modulated radiation therapy (IMRT) Int J Radiat Oncol Biol Phys 2004 60 1 S456 10.1016/j.ijrobp.2004.07.364

